# Nitric Acid and Gum Camphor in the Treatment of Chancroid

**Published:** 1896-10

**Authors:** Lucien Lofton

**Affiliations:** Assistant to the Chair of Anatomy, and Assistant Demonstrator of Anatomy, Southern Medical College, Atlanta, Ga.


### NITRIC ACID AND GUM CAMPHOR IN THE TREATMENT OF CHANCROID.

By LUCIEN LOFTON, M.D.,

Assistant to the Chair of Anatomy, and Assistant Demonstrator of Anatomy, Southern Medical College, Atlanta, Ga.

The various methods for treating chancroidal sores are all more or less painful, unless you have secured local or general anesthesia. To render a sore of this nature free from pain, it is necessary to use an unusual per cent. of cocaine direct to the part, or inject the part freely with a four per cent. solution of the drug mentioned. The danger here lies in new infection. Chancroid is a dangerous and treacherous lesion, and many times the virus plows its way beyond the range of the eye, and when local applications are made this feature of the case should always invariably be looked after. The different mineral acids are used as an escharotic. Carbolic acid is preferred by many, on account of its anesthetic properties. The use of the thermo-cautery and white heat are almost a relic of the past, and I might say a barbarous one.

The main feature in the treatment of this trouble is thorough cauterization and cleanliness.

An application, that I have used with eminent success, both from the standpoint of being painless and thorough, is equal parts by weight of pure nitric acid and camphor gum. Owing to the explosive properties of this compound, it is necessary to always make a fresh mixture. If you do not desire this, by leaving the bottle which contains the solution uncorked, you can keep the compound in good shape indefinitely.

By applying the preparation with a piece of absorbent cotton, wound about a toothpick or match, direct to the sore, little if any pain is experienced. The escharotic effect of the nitric acid is maintained, and the patient is always delighted with the result, should he have experienced the pure acid before.

This same compound acts with equal reliability upon, and in the treatment of, old ulcers and unhealthy granulations.

Just what nitric acid and gum camphor form I am unable to say.

